# Elemental Composition of *Adansonia digitata* L. Bark from Two Sudanese Regions: Effects of Sample Preparation and Preliminary Safety Screening

**DOI:** 10.3390/jox16040147

**Published:** 2026-08-10

**Authors:** Abdelhakam Esmaeil Mohamed Ahmed, Abdaljbbar B. A. Dawod, Eltayeb Omaima Awad Mustafa, Ismail H. Abdi, Elshafia Ali Hamid Mohammed, Béla Kovács

**Affiliations:** 1 Faculty of Agriculture, Food Sciences and Environmental Management, University of Debrecen, Böszörményi Str. 138, 4032 Debrecen, Hungary; elshafia@agr.unideb.hu (E.A.H.M.); kovacsb@agr.unideb.hu (B.K.); 2 Faculty of Forestry, University of Khartoum, Khartoum North 13314, Sudan; 3 Faculty of Informatics, University of Debrecen, Kassai Str. 26, 4028 Debrecen, Hungary; 4 Federal Ministry of Health, Osman Digna Street, P.O. Box 303, Khartoum 11111, Sudan; omaimaawad41@gmail.com; 5 Sudan Medical Specialization Board, Isbitalia Street, Khartoum 13315, Sudan; 6 Department of Mathematics and Natural Sciences, Garissa University, Garissa P.O. Box 1801-70100, Kenya; ismail.abdi@gau.ac.ke; 7 Integrated Pest Management Research Center, Agricultural Research Corporation, Wad Madani 21111, Sudan

**Keywords:** *Adansonia digitata*, baobab bark, elemental composition, ICP-OES, regional variation, bark fractions, sample preparation, trace elements, potentially toxic elements, elemental safety screening

## Abstract

Growing interest in underutilized plant resources has increased the need for comprehensive characterization of their elemental composition. This study investigated the elemental composition of *Adansonia digitata* L. bark collected from two ecologically distinct regions of Sudan and compared elemental distributions among three bark sample forms: whole, cryogenically ground bark (Bark-N), mechanically separated powder (Bark-P), and fibre fraction (Bark-F). Following HNO_3_–H_2_O_2_ wet digestion, elemental concentrations were determined by inductively coupled plasma optical emission spectrometry (ICP-OES). Generalized linear mixed-effects models revealed significant differences in regional and bark sample forms for several essential macroelements, essential trace elements, and other naturally occurring elements. Samples from the Blue Nile region contained higher concentrations of K, P, Fe, Cu, Co, and Mo, whereas bark from North Kordofan showed higher Mg, Na, Mn, Zn, Ba, and Sr concentrations. Distinct elemental profiles were also observed among the three bark sample forms. Selected potentially toxic elements (Pb, Cd, Cr, and As) were detected at low concentrations, providing baseline data for preliminary elemental safety screening. In general, this study provides one of the first comprehensive elemental characterizations of Sudanese baobab bark and establishes a reference dataset for future compositional, pharmaceutical, and industrial research.

## 1. Introduction

**Figure 1:**
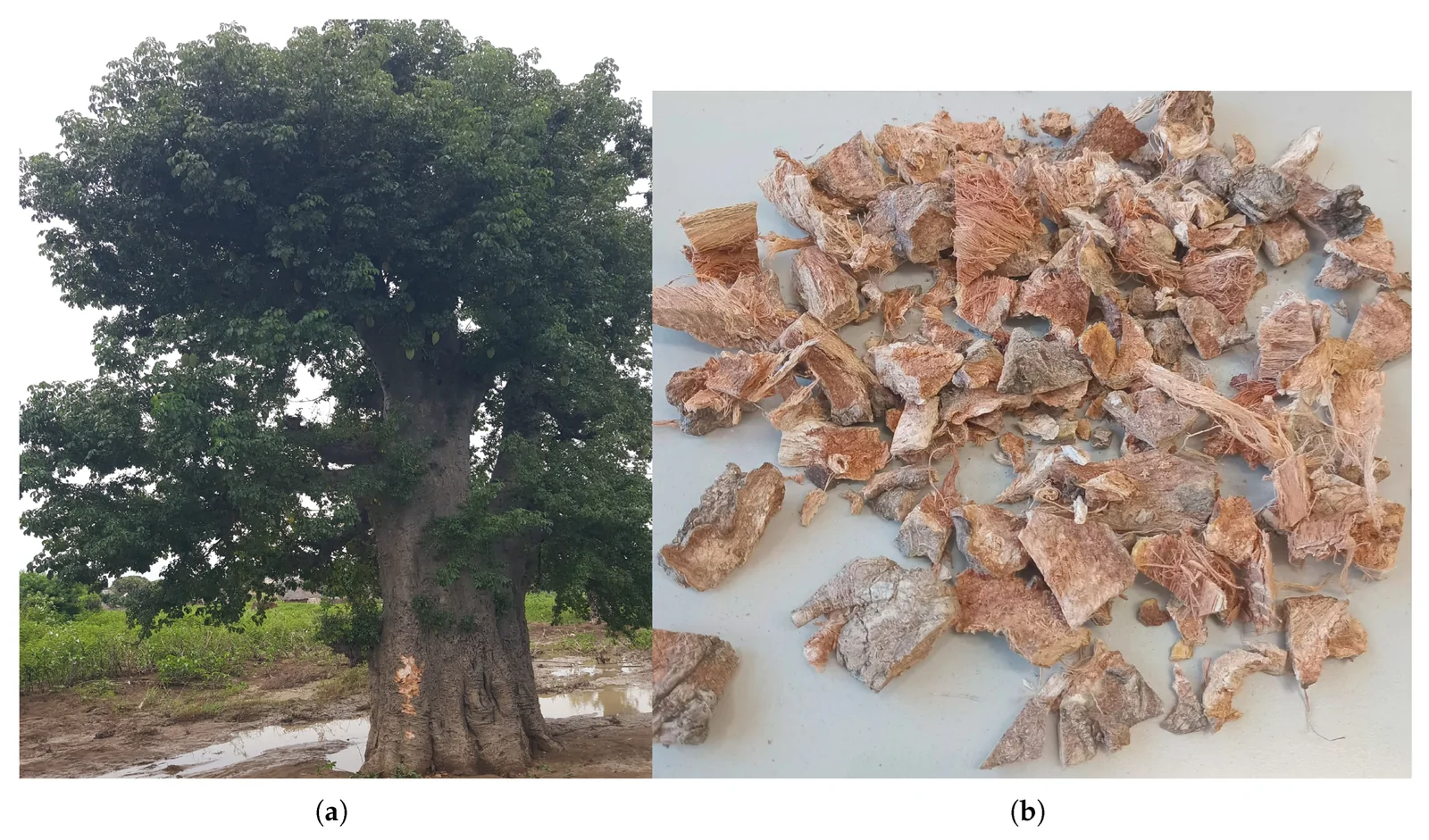
(**a**) Baobab tree found in the Blue Nile region, Sudan; (**b**) Baobab dried bark.

The growing global demand for sustainable plant-based dietary resources has increased the exploration of underutilized species, particularly in the context of food security [1,2], nutrition, functional food development, and sustainable food systems [3,4]. However, alongside their nutritional potential, international concern has increased about the safety and quality of plant-derived foods, particularly regarding the presence of potentially toxic elements [5]. In this regard, baobab (*Adansonia digitata* L.) is a characteristic tree (Figure 1a) of African lands and has long been revered for its multiple uses, traditionally considered an important source of food, fodder, and medicine, as well as for its role in nutritional practices [6,7]. Although the fresh and dried fruit pulp and leaves of *Adansonia digitata* have been extensively investigated for their nutritional composition and health-promoting properties, the bark remains comparatively underexplored despite its long history of use in traditional African medicine. Bark preparations, including decoctions and extracts, have traditionally been used to manage fever, diarrhoea, dysentery, malaria, microbial infections, inflammatory disorders, and wound healing, but do not demonstrate suitability or safety for regular dietary consumption [8]. Therefore, ethnomedicinal applications have stimulated considerable scientific interest in the phytochemical composition and pharmacological properties of baobab bark extracts, which have demonstrated antimicrobial, antioxidant, anti-inflammatory, analgesic, antidiabetic, hepatoprotective, and immunomodulatory activities in experimental studies. Despite the growing body of pharmacological research, relatively little information is available regarding the elemental composition of baobab bark and the distribution of mineral elements among different bark fractions. Characterising its elemental profile is therefore important for improving our understanding of its chemical composition and providing a scientific basis for future nutritional, pharmaceutical, and industrial investigations, without implying suitability for regular dietary consumption [9].

Essential macronutrients such as calcium (Ca), potassium (K), sodium (Na), magnesium (Mg), phosphorus (P), and sulfur (S) are required in relatively large amounts and support physiological functions including bone formation, nerve signaling, electrolyte balance, and metabolic regulation [10]. Trace elements like iron (Fe), manganese (Mn), zinc (Zn), copper (Cu), and selenium (Se), as well as some rare microelements, contribute to the regulation of oxidative stress, antioxidant defense, immune function, enzymatic activity, and blood formation in the body. These minerals strongly influence the nutritional value and functional characteristics of many plant-based products [11].

In addition to essential mineral nutrients, plants naturally contain a range of other elements, including boron (B), strontium (Sr), barium (Ba), nickel (Ni), titanium (Ti), vanadium (V), and aluminum (Al). These elements may occur naturally in plant tissues due to species-specific uptake and environmental conditions. Although some have been reported to possess biological or physiological relevance under specific conditions, most are not recognized as essential nutrients for humans. Therefore, characterizing their occurrence contributes to a more comprehensive understanding of the elemental composition of baobab bark rather than implying nutritional benefit [12]. On the other hand, the presence of potentially toxic elements such as lead (Pb), cadmium (Cd), chromium (Cr), and arsenic (As) raises safety concerns regarding their use in nutrition [13]. Evaluating all four elemental categories is necessary to characterise the elemental profile and provide preliminary information relevant to future safety evaluation.

Elemental composition also varies with ecological conditions [14]. Sudan covers numerous environmental regions, and baobab bark collected from diverse areas may vary noticeably in mineral accumulation level; however, its mineral composition and safety aspects remain insufficiently documented. Another critical factor in chemical analysis is sample preparation. Baobab bark is fibrous and difficult to grind, and sample preparation (sample preparation), such as liquid-nitrogen milling [15], of semisolid plant tissues as a mechanical powdering method may affect its elemental assessment.

Previous studies have primarily focused on the phytochemical and medicinal properties of baobab bark, whereas its elemental composition and the influence of sample preparation on elemental distribution remain insufficiently characterized [16]. A comprehensive characterisation of the elemental profile of baobab bark collected from different regions may improve understanding of its composition and support future investigations into its potential applications as a plant-derived resource. The present study therefore aimed to characterize the elemental composition of *Adansonia digitata* bark (Figure 1b) collected from two regions of Sudan and to examine differences among three bark sample forms produced by cryogenic grinding (Bark-N) and mechanical fractionation (Bark-P and Bark-F). Specifically, the objectives were: (i) to quantify the concentrations of essential macroelements, essential trace elements, other naturally occurring elements, and selected potentially toxic elements in baobab bark; (ii) to compare elemental composition between bark samples collected from the Blue Nile and North Kordofan regions; (iii) to evaluate differences in elemental distribution among whole bark, powder, and fibre fractions obtained during sample preparation; and (iv) to provide a preliminary elemental safety screening based on the measured concentrations of selected potentially toxic elements.

## 2. Materials and Methods

### 2.1. Samples Collection

**Figure 2:**
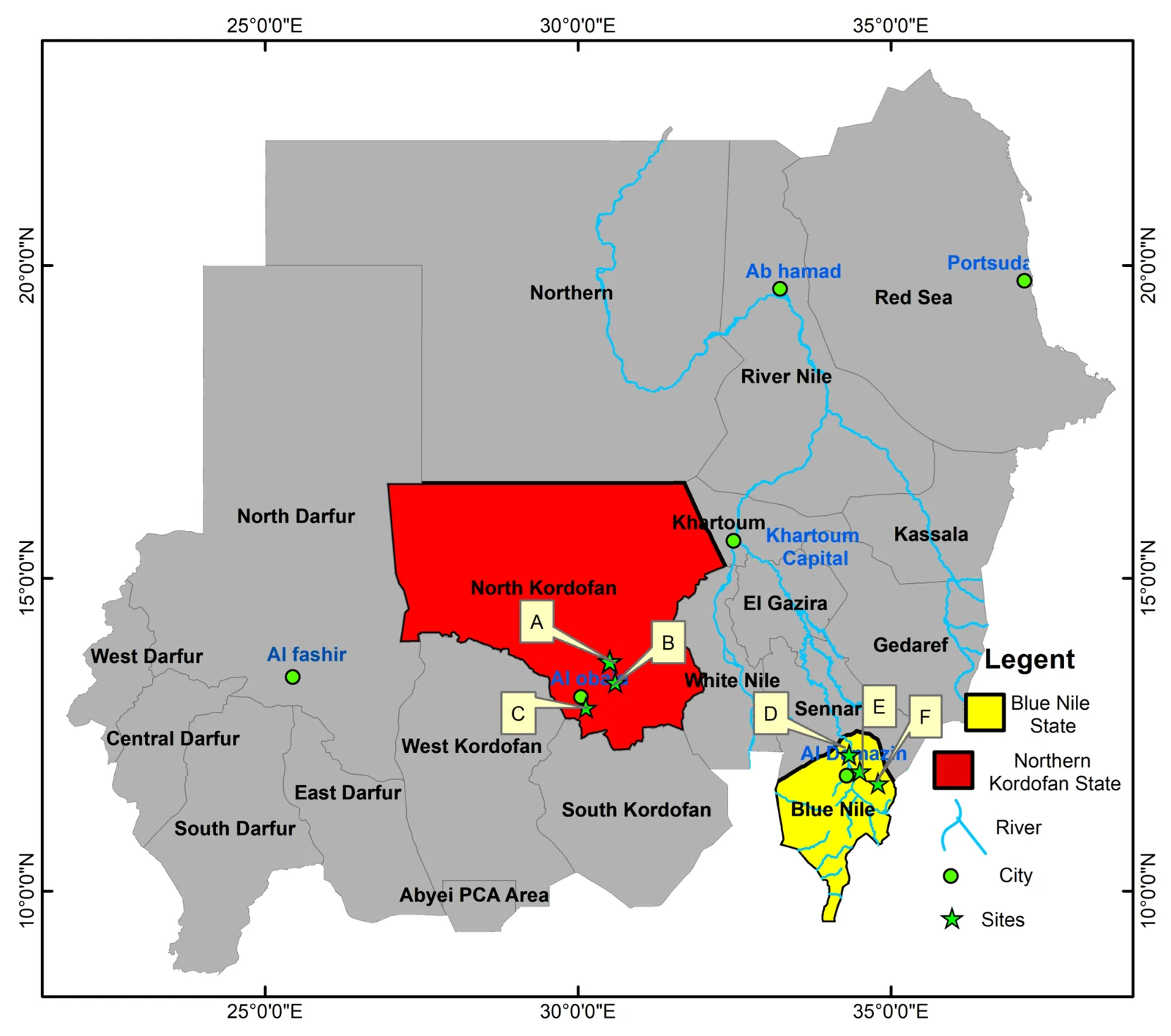
Location of baobab bark sample collections: North Kordofan and the Blue Nile in Sudan. Blocks A–C represent the three sampling blocks in North Kordofan, whereas Blocks D–F represent the three sampling blocks in the Blue Nile region.

For this preliminary study, Baobab bark sampling was conducted in Sudan between 15 July 2022 and 15 September 2022. Baobab (*Adansonia digitata* L.) bark samples were collected from two major baobab- growing regions: the Blue Nile region (Latitude: 11°15′00′′ N; Longitude: 34°10′00′′ E) represents a relatively wet environment and the North Kordofan region (approximately between Latitude: 12°40′ N–14°20′ N and Longitude: 28°10′ E–31°40′ E), representing a dry, semi-arid climate (Figure 2). Three blocks were chosen per region: A, B, and C in Kordofan, and D, E, and F in the Blue Nile; see Figure 2. Each selected block covered approximately 1500 km^2^. The study area map was generated using QGIS software (version 3.20.1). The bark layer samples were collected from the selected trees at random heights ranging from 70 cm to 1.5 m. Thirty mature and apparently healthy baobab trees were randomly selected within each region (10 trees per block). There were 60 independent trees in total, with three repeated sample-form observations per tree. Bark collected from each tree was processed into three bark sample forms (Bark-N, Bark-P and Bark-F), which were analysed as repeated measurements from the same biological replicate. Three sampling blocks were selected within each region to represent the regional variability. As the blocks were intended to characterise the respective regions, they were not modelled as an independent random effect. Instead, tree identity was included as a random intercept to account for repeated measurements from the same biological replicate. At the same time, region, bark sample form, and their interaction were specified as fixed effects in the linear mixed-effects model. The collected bark samples were shade-dried to preserve their natural characteristics and then transported to the Institute of Food Science at the University of Debrecen (Hungary) for further processing and elemental analysis. The bark samples collected from each selected tree were manually cleaned to remove visible surface contaminants before sample preparation. Each bark sample was processed individually into three sample forms: (i) whole cryogenically ground bark (Bark-N), (ii) powder fraction obtained by mechanical separation (Bark-P), and (iii) fibre-rich fraction remaining after mechanical separation (Bark-F). The elemental composition of these three bark sample forms was subsequently determined to evaluate the distribution of mineral elements among the different bark fractions.

### 2.2. Preparation and Wet Digestion

The bark samples collected from each selected tree were manually cleaned to remove visible surface contaminants before sample preparation. The plant samples were dried at 105 °C to a constant weight before being ground to a fine powder. The stainless-steel electric grinder is equipped with a 10 mm sieve and is constructed from a material that differs from the analytes of interest to prevent potential contamination by Fe, Cr, Ni, or Mn during milling. The stainless-steel grinding components were selected to ensure efficient sample homogenisation and minimise potential contamination during sample preparation.

Each bark sample was processed individually into three sample forms: (i) whole cryogenically ground bark using liquid nitrogen (N_2_), then named (Bark-N), (ii) powder fraction obtained by mechanical separation (Bark-P), and (iii) fibre-rich fraction remaining after mechanical separation (Bark-F). Approximately 2 g of the dried sample was weighed into the digestion tubes. Wet digestion was carried out using a nitric acid–hydrogen peroxide HNO_3_-H_2_O_2_ method. Ten millilitres of concentrated HNO_3_ (65%) was added to each sample. Predigestion was performed at 60 °C for 30 min or overnight at room temperature. After predigestion, 3 mL of 30% H_2_O_2_ was added. The samples were then digested at 120 °C for 90 min using a block digestion system as described by [17]. After digestion, the solutions were cooled and diluted to a final volume of 50 mL with ultrapure water. The digested solutions were filtered through filter paper before analysis.

### 2.3. Mineral Analysis

Elemental analysis of dried baobab bark samples was performed using inductively coupled plasma optical emission spectrometry (ICP-OES) using a peristaltic pump system. A specific ICP-OES spectrometer (iCAP 6300) manufactured by Thermo Fisher Scientific (Waltham, MA, USA) was used for this analysis. The instrument was operated under optimized conditions. The viewing height was set at 5 mm. The forward power was 1000 W. The sample gas flow rate was 1.14 L min^−1^. The coolant gas flow rate was 10 L min^−1^. The auxiliary gas flow rate was 0.1 L min^−1^. The flushing gas flow rate was 0.13 L min^−1^, the radio-frequency power was 1200 W, and the viewing height was 5 mm. The sample uptake rate was 4 mL min^−1^. Calibration standards were prepared using standard solutions produced by BDH Chemicals Ltd. (Poole, England) and Merck GmbH (Darmstadt, Germany), as well as pro-analytical-grade solid chemicals from REANAL Ltd. (Budapest, Hungary). The analytical procedure and instrumental parameters applied were in accordance with [17] and are aligned with protocols validated by the International Plant-Analytical Exchange (IPE-245) proficiency testing scheme coordinated by Wageningen Evaluating Programs for Analytical Laboratories (WEPAL) [18,19]. This scheme uses unmodified natural reference materials, such as Salix alba (willow wood), to establish consensus values through inter-laboratory comparisons and to benchmark analytical quality. Quantification of mineral elements, including Ca, Mg, K, Na, Fe, and Mn, was performed on the acid-digested solution. The acid extract was used to quantify the analysed elements, which were expressed as parts per million (ppm) on a dry matter (DM) basis. For more understanding, analysed elements were classified into four categories: essential macro-elements (Ca, K, Na, Mg, P, S), essential trace elements (Fe, Mn, Zn, Cu, Co, Mo), other naturally occurring elements (B, Ba, Sr, Ni, Ti, V, Al) and potential toxic elements (Pb, Cd, Cr, As). Three instrumental (technical) replicate measurements were performed for each prepared sample to assess analytical repeatability. The technical replicates were averaged before statistical analysis.

### 2.4. Quality Assurance and Quality Control (QA/QC)

Calibration of the ICP-OES used was performed using certified mono-element and multi-element standard solutions. Analytical quality assurance included evaluation of calibration linearity (R^2^), analytical wavelengths, calibration ranges, limits of detection (LOD), limits of quantification (LOQ), procedural blanks, certified reference material (CRM) validation, and instrument stability. Procedural blanks, including calibration blanks and digestion blanks, were analysed throughout the analytical sequence, and all blank concentrations were below the respective limits of detection, indicating negligible contamination during sample preparation and analysis. Instrument stability was verified by analysing a mid-level calibration standard after completion of the sample sequence, and no significant calibration drift was observed. Analytical accuracy was assessed using the certified reference material ERM^®^-CD281 [20], for elements with certified concentrations and assigned consensus reference values from the International Plant-Analytical Exchange (IPE/WEPAL) proficiency testing scheme for elements without certified CRM values [19]. Recoveries ranged from 90.37% to 108.00%, demonstrating satisfactory analytical accuracy. Recovery was not evaluated for Ti because no certified or assigned reference value was available, whereas As and Cd were below the laboratory reporting limits during validation. Detailed QA/QC parameters and validation results are provided in the Appendix A Table A1 and Table A2.

### 2.5. Statistical Analysis

**Figure 3:**
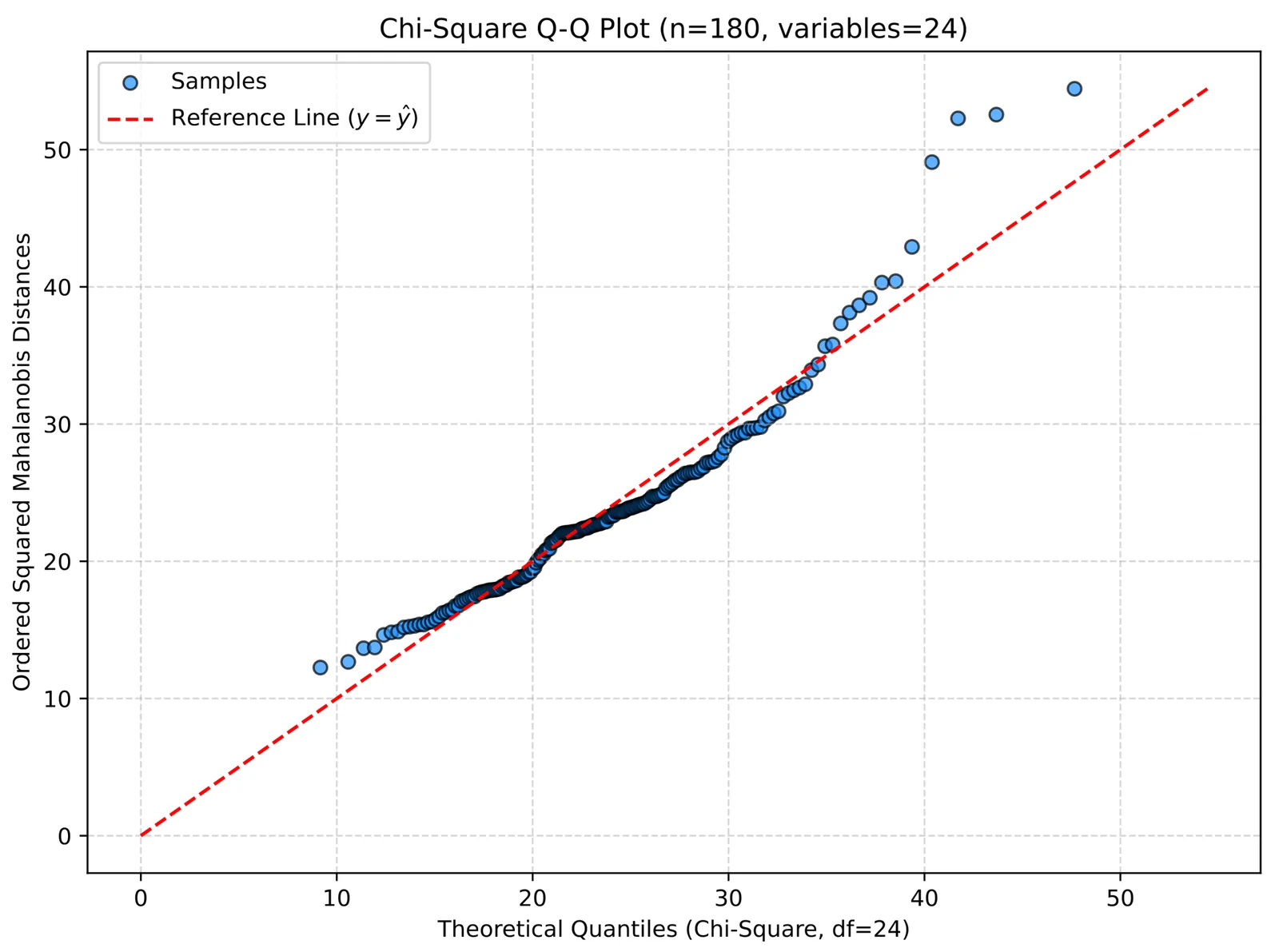
Multivariate quantile–quantile (Q–Q) plot based on Mahalanobis distances to assess multivariate normality.

Multivariate normality of the elemental concentration data was assessed using Mardia’s test. The results indicated a significant departure from multivariate normality, with Mardia’s skewness statistic of 256.00 (p<0.001), whereas the kurtosis statistic (629.03, p=0.339) did not differ significantly from that expected under a multivariate normal distribution. These findings suggest that the violation of multivariate normality was primarily attributable to multivariate skewness rather than excessive kurtosis. Consistent with these results, the QQ plot in Figure 3 and the Shapiro–Wilk test also indicated significant deviations from normality. Accordingly, descriptive data are expressed as the median and interquartile range (IQR).

To satisfy the assumption of normally distributed residuals required by linear mixed-effects models (LMMs), the continuous response variables representing elemental concentrations were first subjected to a rank-based inverse normal transformation (INT). Raw measurements were converted to ascending ranks, scaled to fractional probabilities using Blom’s continuity correction—calculated as $(r_{i}-0.5)/N$, where $r_{i}$ is the rank of the *i*-th observation and *N* is the total sample size—and mapped to standard normal *Z*-scores via the inverse cumulative distribution function.

The normalized data were subsequently analyzed using LMMs configured with a Gaussian error distribution and an identity link function. To systematically evaluate the influences of geographical origin and bark sample forms while robustly accounting for the non-independence of repeated measures, models were structured with Region and Bark Sample Form as fixed effects, alongside the individual Tree as a random intercept. Treatment contrast coding was applied, designating *Blue Nile* and *Bark-N* as the a priori reference categories for region and sample form, respectively. Consequently, the estimated fixed-effect coefficients provide a direct, standardized measure (in standard deviation units) quantifying the shift in elemental recovery relative to these geographical and methodological baselines.

The overall statistical significance of the fixed effects and their interactions was assessed using likelihood ratio tests. To elucidate significant differences between specific sample forms, post hoc pairwise comparisons were conducted. To strictly control the family-wise error rate inflation inherent in multiple testing, all pairwise *p*-values were adjusted using the Holm step-down procedure. Finally, isolating the variance component of the random intercept enabled the models to partition total variance, differentiating compositional alterations driven by regional and mechanical factors from the inherent biological variation among individual trees.

Model parameters were estimated using Restricted Maximum Likelihood (REML) to ensure unbiased estimates of the variance components. All data transformations and statistical modeling were conducted in Python (version 3.11), utilising the *scipy.stats* library for the INT procedure and the *statsmodels* package (v0.14) for mixed-model estimation. Model adequacy was evaluated through residual diagnostics, including histograms and QQ plots for visual assessment of normality, the Shapiro–Wilk test for residual normality, and residual-versus-tree plots to verify homoscedasticity and the appropriateness of the random-effects structure.

Associations among elemental concentrations were assessed using Spearman’s rank correlation coefficient because of its robustness to non-normality and outliers. Principal component analysis (PCA) was used to visualize patterns in elemental composition. All statistical analyses and computational procedures were performed in Python (version 3.12.13) using the Google Colab environment.

## 3. Results

Initial comparisons of elemental concentrations identified differences among bark sample forms within each region using non-parametric tests. Subsequently, linear mixed-effects models quantified the independent and interactive effects of region and bark sample forms while accounting for repeated measurements from the same tree. Significant interaction effects were observed through the Likelihood ratio tests. This approach enabled a comprehensive assessment of both the main effects and their interaction on the elemental composition of baobab bark. In addition, the elemental correlation heatmap shows a complex network of identified nutrients in each region, and the PCA results were used to visualise both regions and bark sample forms. All results were presented as follows:

### 3.1. Regional Variability in Elemental Composition

**Figure 4:**
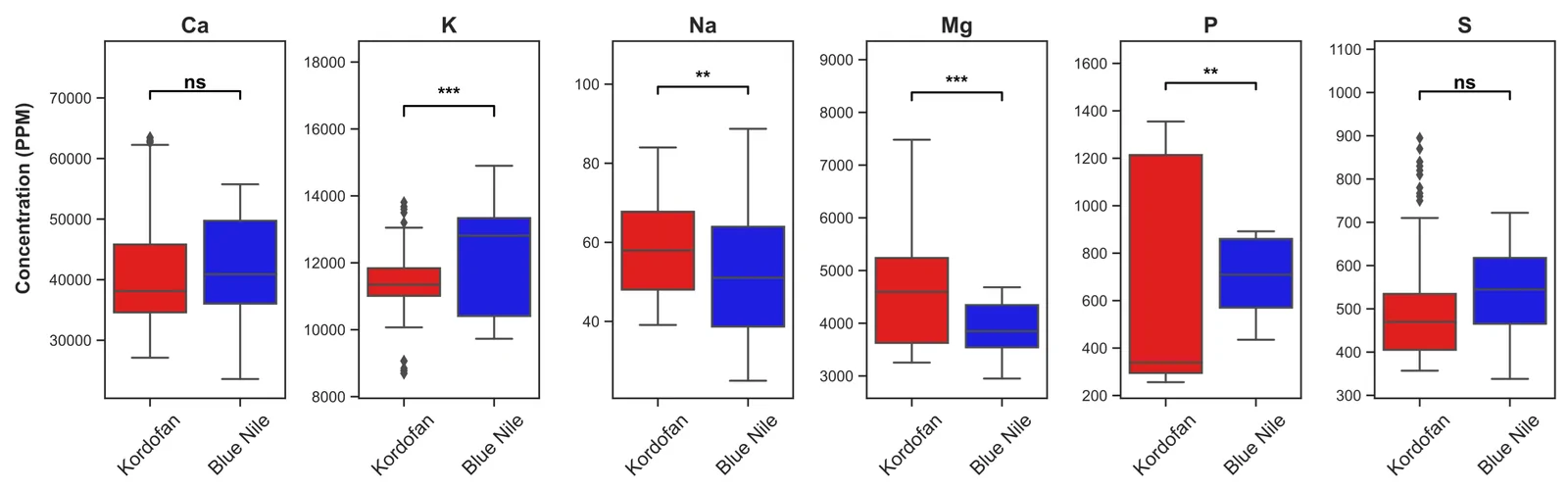
Regional variation in the concentrations of essential macroelements (Ca, K, Na, Mg, P, and S) in baobab bark collected from the Blue Nile and Kordofan regions of Sudan (*n* = 60). Boxplots display the median (horizontal line) (Q1–Q3; box) and range (whiskers). Regional differences were evaluated using LMMs. Significance levels are denoted as ns, not significant; ** p<0.01; *** p<0.001.

**Figure 5:**
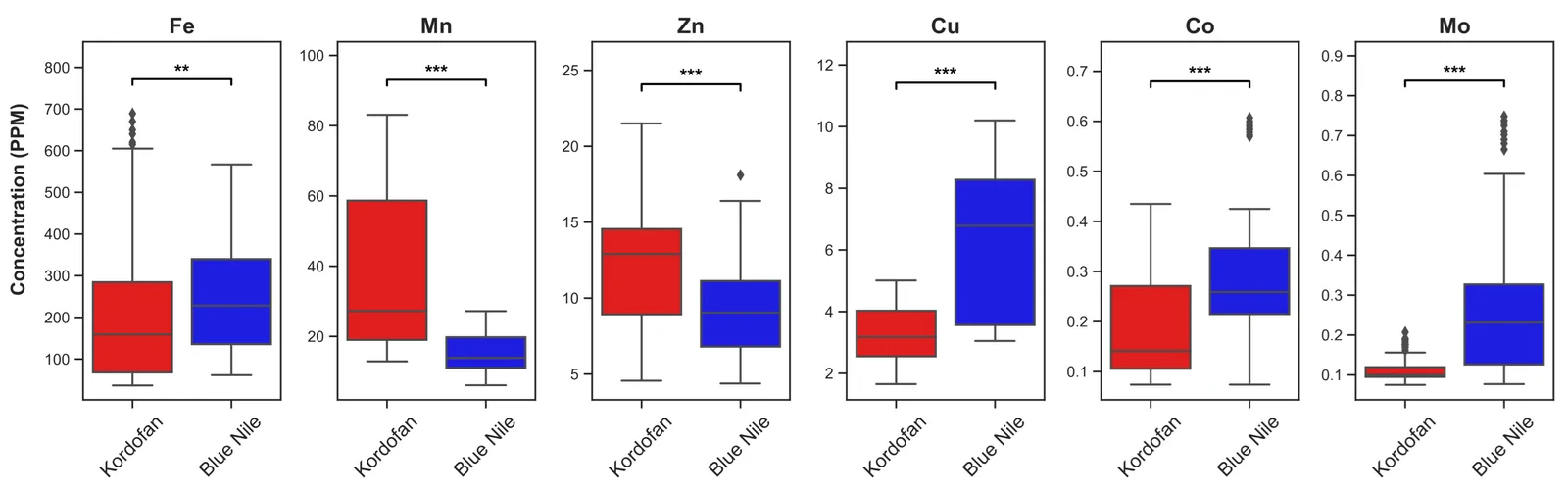
Regional variation in the concentration of the essential trace elements (Fe, Cu, Mn, Zn, Co, and Mo) in baobab bark collected from the Blue Nile and Kordofan regions of Sudan (*n* = 60). Boxplots display the median (horizontal line) (Q1–Q3; box) and range (whiskers). Regional differences were evaluated using LMMs. Significance levels are denoted as ** p<0.01; *** p<0.001.

**Figure 6:**
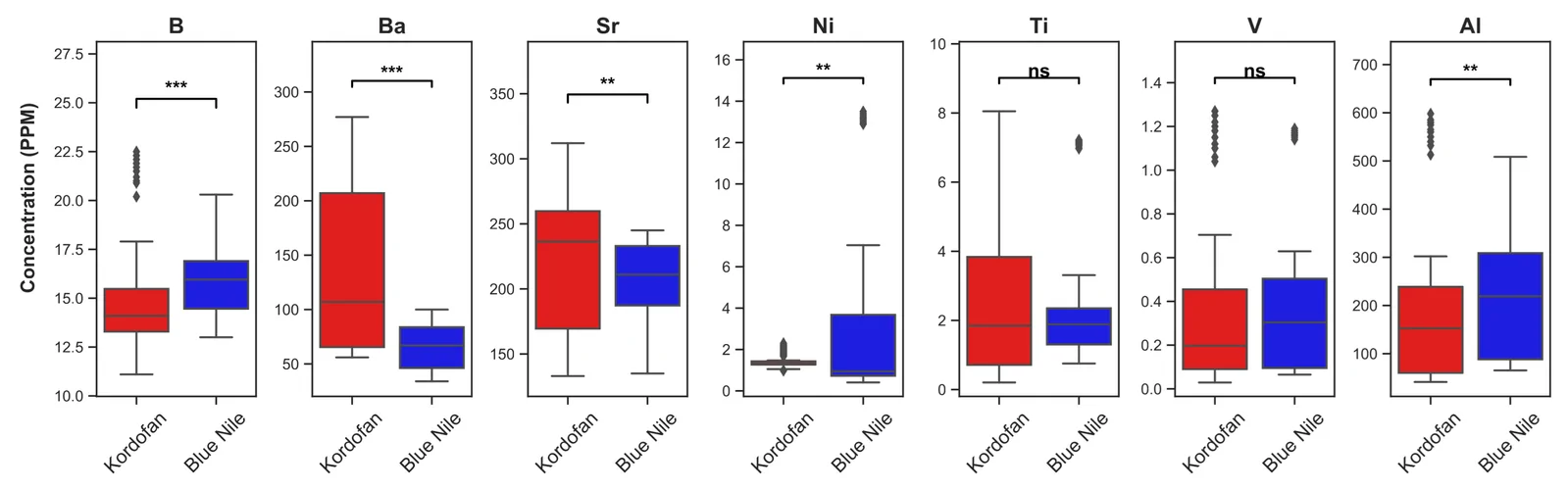
Regional variation in the concentrations of other naturally occurring elements (B, Ba, Sr, Ni, Ti, V, and Al) in baobab bark collected from the Blue Nile and Kordofan regions of Sudan (*n* = 60). Boxplots display the median (horizontal line) (Q1–Q3; box) and range (whiskers). Regional differences were evaluated using LMMs. Significance levels are denoted as ns, not significant; ** p<0.01; *** p<0.001.

**Figure 7:**
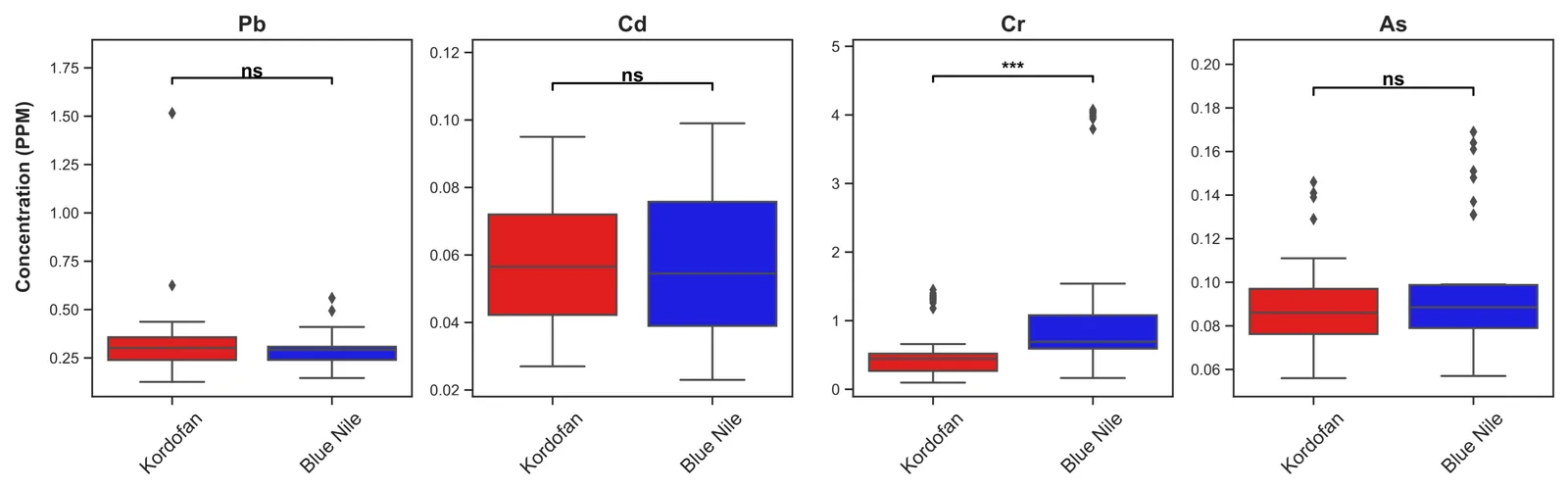
Regional variation in the concentrations of naturally occurring elements (B, Ba, Sr, Ni, Ti, V, and Al) in baobab bark collected from the Blue Nile and Kordofan regions of Sudan (*n* = 60). Boxplots display the median (horizontal line) (Q1–Q3; box) and range (whiskers). Regional differences were evaluated using LMMs. Significance levels are denoted as ns, not significant; *** p<0.001.

The elemental composition of baobab bark differed between the Blue Nile and Kordofan regions (Table 1; Figure 4, Figure 5, Figure 6 and Figure 7). Among the essential macroelements, the Blue Nile samples showed higher median concentrations of Ca (40,900.0 ppm), K (12,810.0 ppm), P (709.5 ppm), and S (545.0 ppm), whereas Mg (4596.5 ppm) and Na (57.95 ppm) were higher in the Kordofan samples (Table 1). Statistical analysis indicated significant regional differences for K, Na, Mg, and P, whereas Ca and S did not differ significantly between the two regions (Figure 4).

Distinct regional patterns were also observed for the essential trace elements (Figure 5). The Blue Nile samples contained higher median concentrations of Fe (228.0 ppm), Cu (6.79 ppm), Co (0.26 ppm), and Mo (0.23 ppm), while Mn (27.24 ppm) and Zn (12.90 ppm) were more abundant in the North Kordofan samples. All six trace elements showed significant regional differences, with the largest separation observed for Mn, Zn, Cu, Co, and Mo.

For the other naturally occurring elements (Figure 6), B, Ni, and Al were more abundant in the Blue Nile samples, whereas Ba and Sr showed higher median concentrations in the North Kordofan samples. In contrast, Ti and V exhibited comparable concentrations between the two regions and showed no significant regional differences.

The concentrations of selected potentially toxic elements were generally similar between regions (Figure 7). Chromium showed a significantly higher concentration in the Blue Nile samples, whereas Pb, Cd, and As exhibited only minor regional variation and did not differ significantly between the two regions.

The boxplots further illustrate the regional variability in elemental composition. Several elements, including K, Mg, Mn, Zn, Cu, Co, Mo, B, Ba, and Cr, exhibited clear separation between regional distributions with limited overlap of the interquartile ranges, whereas Ca, S, Ti, V, Pb, Cd, and As showed substantial overlap, indicating comparable concentrations between the two sampled regions.

The results demonstrate that the two regional composite bark samples differed in the distribution of several elements, while the concentrations of most potentially toxic elements remained comparable.

### 3.2. Elemental Distribution Among Bark Sample Forms

**Figure 8:**
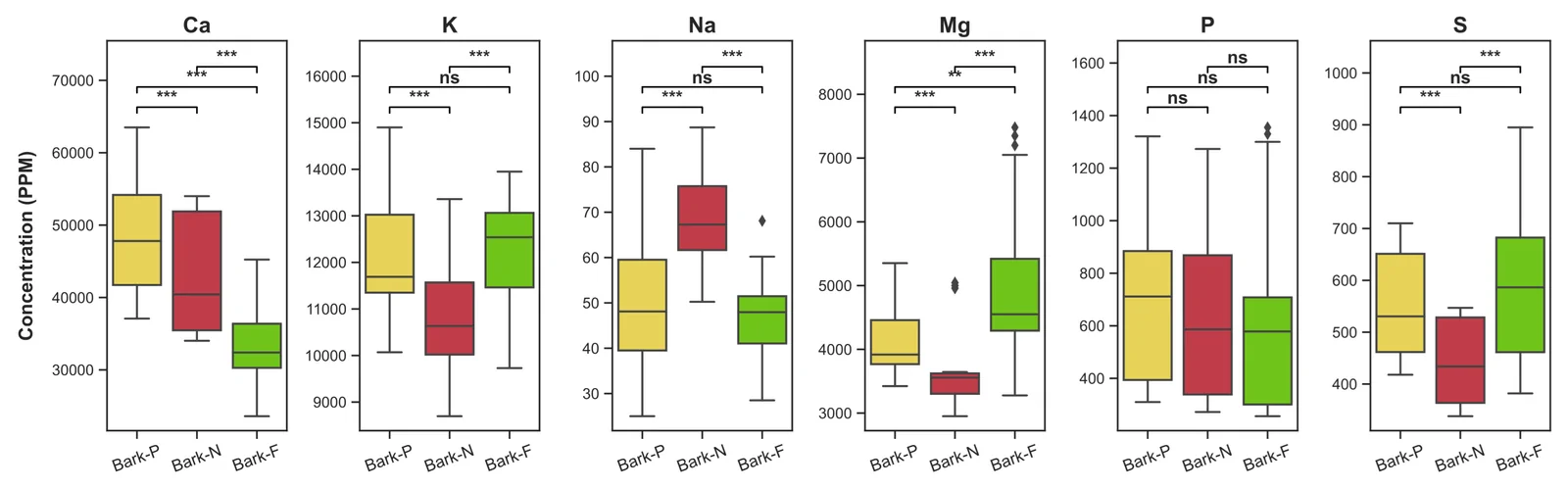
Variations in essential macroelement concentrations (Ca, K, Na, Mg, P, and S) among baobab bark sample forms (*n* = 60). Fractions include whole cryogenically ground bark (Bark-N), mechanically separated fibre (Bark-F), and powder (Bark-P). Pairwise differences were assessed via LMM. Significance levels are indicated as follows: ns, not significant; ** p<0.01; *** p<0.001.

**Figure 9:**
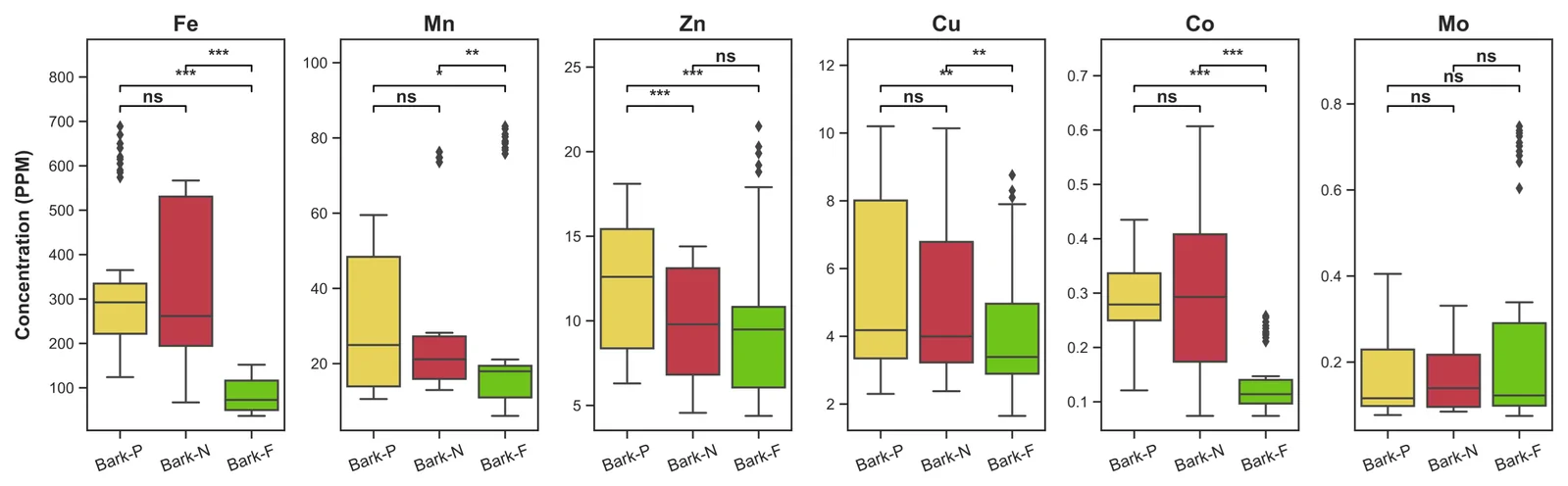
Variations in essential trace element concentrations (Fe, Cu, Mn, Zn, Co, and Mo) among the three baobab bark sample forms (*n* = 60): Bark-N, Bark-F, and Bark-P. Pairwise comparisons were performed using the LMM test. Significance levels are indicated as follows: ns, not significant; * p<0.05; ** p<0.01; *** p<0.001.

**Figure 10:**
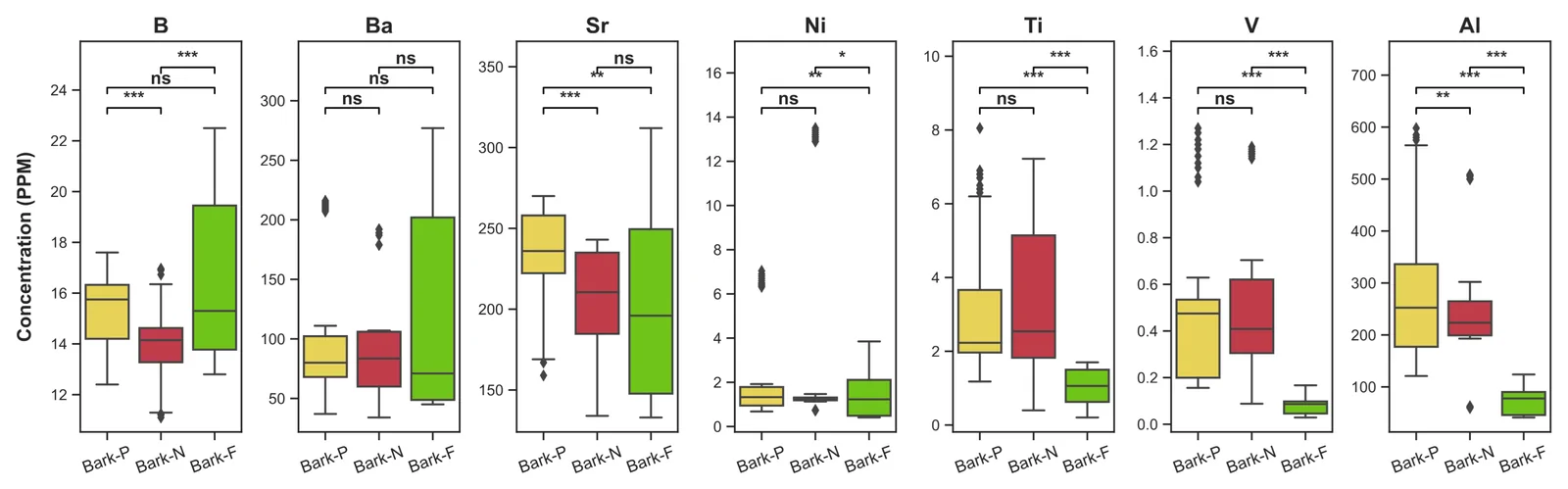
Variations in concentrations of other naturally occurring elements (B, Ba, Sr, Ni, Ti, V, and Al) among the three baobab bark sample forms (*n* = 60): Bark-N, Bark-F, and Bark-P. Pairwise comparisons were performed using the LMM test. Significance levels are indicated as follows: ns, not significant; * p<0.05; ** p<0.01; *** p<0.001.

**Figure 11:**
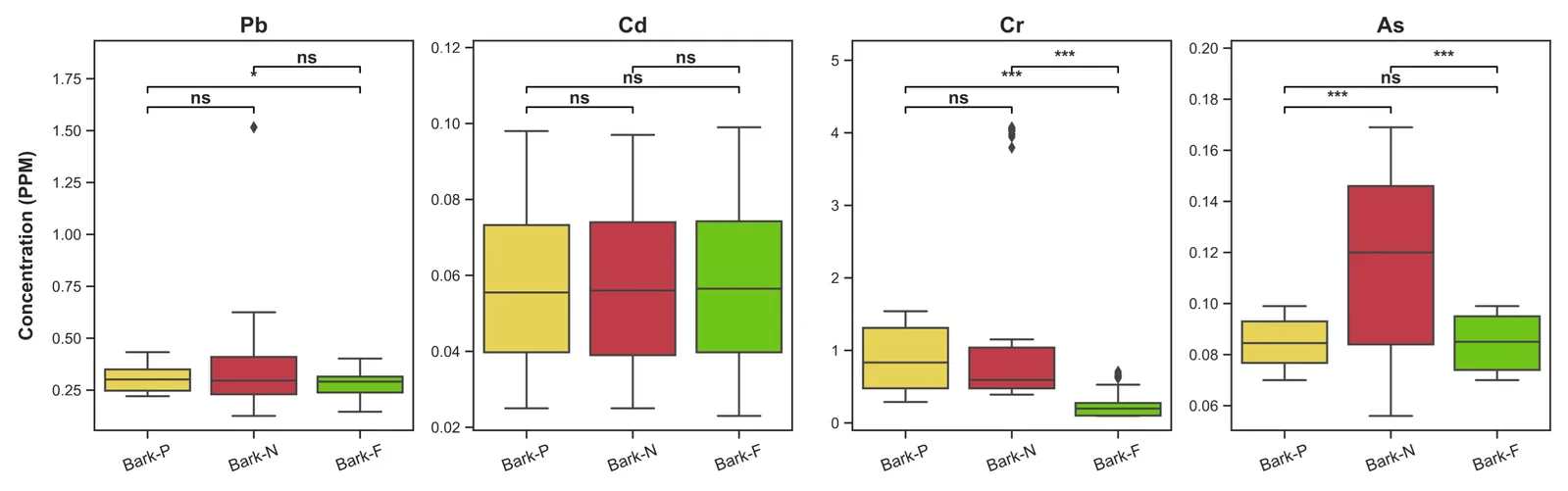
Variations in selected potentially toxic element concentrations (Pb, Cd, Cr, and As) among the three baobab bark sample forms (*n* = 60): Bark-N, Bark-F, and Bark-P. Pairwise comparisons were performed using the LMM test. Significance levels are indicated as follows: ns, not significant; * p<0.05; *** p<0.001.

The elemental composition varied among the three bark sample forms (Table 2; Figure 8, Figure 9, Figure 10 and Figure 11). Among the essential macroelements, the fibre fraction (Bark-F) showed the highest median concentrations of Ca (47,805.0 ppm), Mg (3917.5 ppm), P (711.5 ppm), and S (530.5 ppm), whereas the powder fraction (Bark-P) contained the highest concentrations of K (12,540.0 ppm). Whole cryogenically ground bark (Bark-N) exhibited the highest median Na concentration (67.28 ppm). Pairwise comparisons showed significant differences for most macroelements, whereas P exhibited relatively small variation among the three sample forms (Figure 8).

Clear differences were also observed for the essential trace elements (Figure 9). Bark-F contained the highest median concentrations of Fe (292.5 ppm), Mn (24.95 ppm), Cu (4.18 ppm), and Co (0.28 ppm), whereas Bark-N showed the highest concentrations of Zn (9.79 ppm) and Mo (0.14 ppm). The boxplots indicate that Fe, Zn, Co, and Mo exhibited the greatest separation among sample forms, while Mn and Cu showed greater overlap between Bark-N and Bark-F.

Among the other naturally occurring elements (Figure 10), Bark-F showed the highest median concentrations of B (15.75 ppm), Sr (236.0 ppm), Ni (1.33 ppm), Ti (2.23 ppm), V (0.48 ppm), and Al (252.5 ppm). In contrast, Bark-N contained slightly higher Ba concentrations (83.5 ppm). Several elements, including Ba, Sr, Ti, V, and Al, showed clear differences among bark sample forms, whereas B and Ni exhibited greater overlap between groups.

The concentrations of selected potentially toxic elements also varied among the bark sample forms (Figure 11). Chromium showed the highest median concentration in Bark-F (0.83 ppm), whereas Pb and Cd exhibited similar concentrations across all three sample forms. Arsenic showed only modest variation, with slightly higher concentrations in Bark-N. Significant differences were observed for Cr and As, whereas Pb and Cd remained comparable among the bark sample forms.

Overall, the three bark sample forms exhibited distinct elemental profiles. The observed differences most likely reflect differences in the physical composition of the whole bark, powder, and fibre fractions generated during sample preparation rather than changes in elemental concentrations caused by the grinding process itself. These results describe the distribution of elements among the analysed bark sample forms and provide a basis for further investigation of their compositional characteristics.

### 3.3. Main, Interaction, and Simple Main Effects on Elemental Distribution

To evaluate how regional variability, bark preparation methods, and their combined interaction affect elemental distribution, separate LMMs were fitted for each element. Region, Bark Sample Form, and their interaction term (Region × Sample Form) were specified as fixed effects. The individual tree was included as a random intercept to rigorously account for the non-independence of repeated measurements. The significance of the fixed effects was assessed using likelihood ratio tests with 1, 2, and 2 degrees of freedom, respectively (Table 3). To elucidate significant interactions, the models were subsequently nested to extract pairwise comparisons of sample forms within regions (Table 4) and the simple main effects of region isolated within specific sample forms (Table 5).

Likelihood ratio tests demonstrated that elemental distribution in baobab bark is pervasively governed by both regional variability and sample preparation, frequently in an interactive manner (Table 3). Bark sample form had a significant main effect on most elements (p≤0.011), with the exceptions of P, Mo, Ba, Pb, and Cd. Regional main effects were similarly widespread, though notably absent for Ca, S, Ti, Pb, Cd, and As. Crucially, significant Region × Sample Form interactions were identified for most elements across all elemental groups. The absence of significant interactions for P, S, Cu, Ba, and Cd suggests these specific elements respond consistently to preparation methods regardless of regional differences; however, for the remaining elements, the influence of bark preparation differed substantially between the Blue Nile and North Kordofan regions.

To decompose these interaction effects, intra-regional pairwise comparisons were assessed (Table 4). The transition from Bark-N to Bark-F forms yielded highly significant elemental shifts within both regions. Furthermore, computing the simple main effects of region isolated within specific bark forms revealed highly regional variability (Table 5). For example, while Bark-N and Bark-f exhibited significant regional differences in Ca and Fe, the Bark-P form statistically neutralised these regional variances (p=0.953 and p=0.326, respectively). Conversely, regional divergence for K and Na was pronounced strictly within the Bark-P. These results underscore that regional tracing of baobab bark necessitates tightly controlled, preparation-specific baselines.

### 3.4. Spearman Correlation Analysis

**Figure 12:**
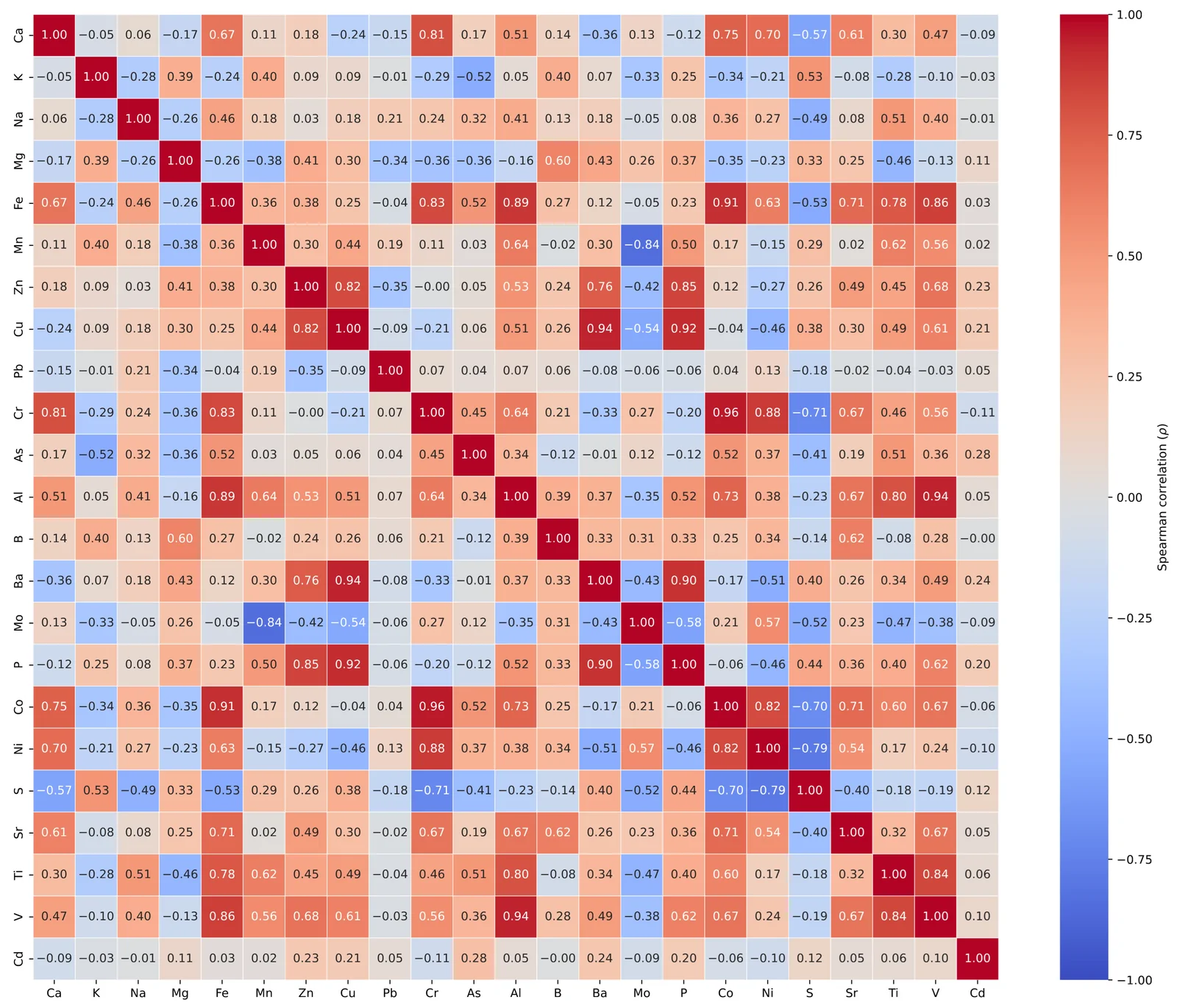
Heatmap displaying Spearman correlation coefficients among the macro-element, trace element, and potentially toxic element concentrations in baobab bark for the Blue Nile region, prepared as three bark sample forms: Bark-N, Bark-F, and Bark-P.

**Figure 13:**
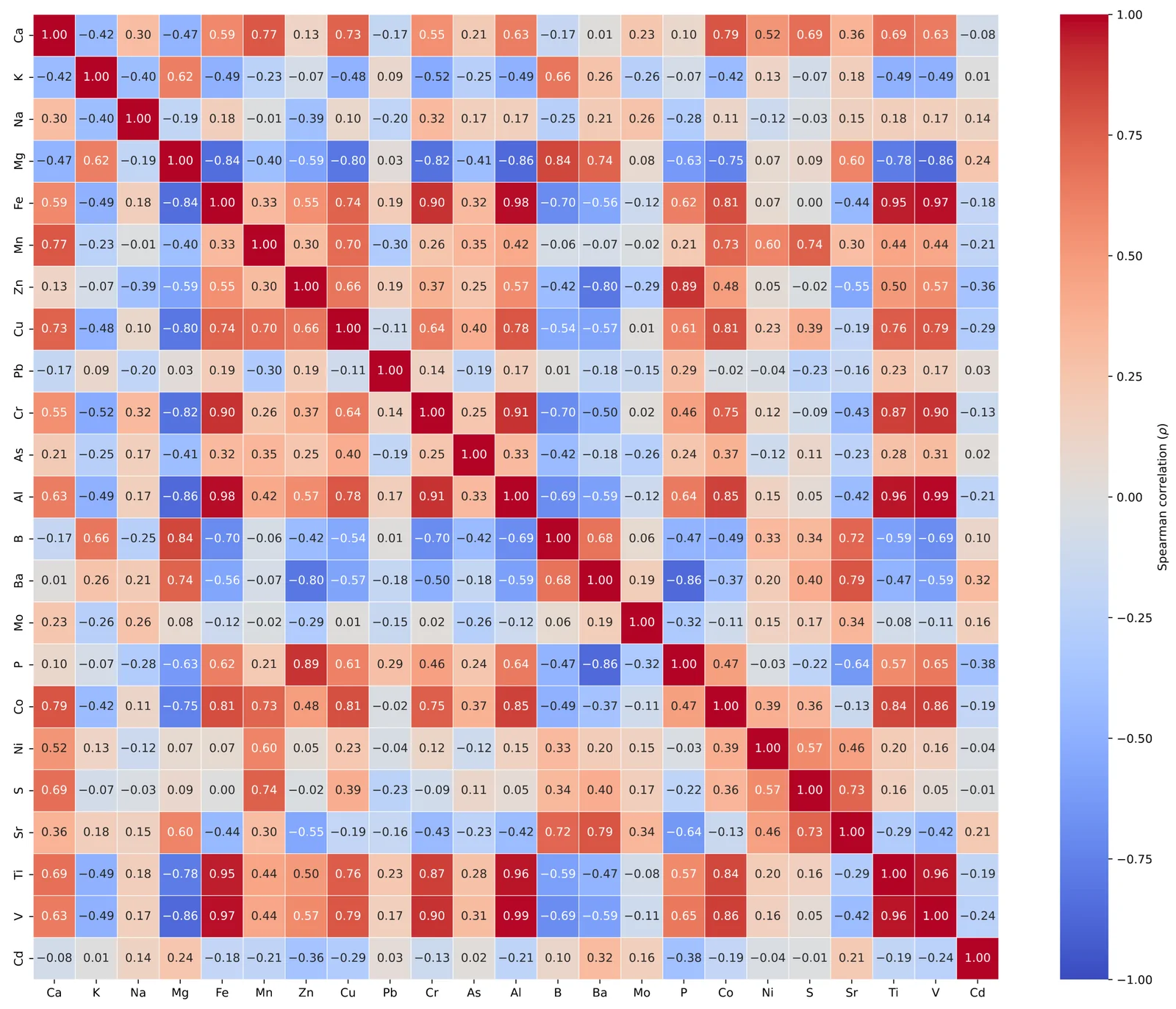
Heatmap showing Spearman correlation coefficients among macro-, trace-, and potentially toxic element concentrations in baobab bark from the Kordofan region, prepared as three bark sample forms: Bark-N, Bark-F, and Bark-P.

The Spearman correlation heatmaps (Figure 12 and Figure 13) revealed distinct patterns of elemental associations in both the Blue Nile and Kordofan regions. Several essential and trace elements exhibited strong positive correlations.

In the Kordofan samples (Figure 13), a strong positive correlation network was observed among Fe, Al, Ti, V, and Cr (r=0.87−0.99), indicating a closely associated group of elements. Similarly, Ca showed strong positive correlations with Mn (r=0.77), Co (r=0.79), S (r=0.69), and Cu (r=0.73), while exhibiting moderate-to-strong negative correlations with Mg (r=−0.47) and K (r=−0.42). Magnesium displayed strong inverse relationships with Fe (r=−0.84), Al (r=−0.86), Ti (r=−0.78), and V (r=−0.86), suggesting contrasting accumulation patterns between these elemental groups. Most heavy metals showed weak correlations with most macro-elements, except for Cr, which clustered strongly with Fe, Al, Ti, and V.

In contrast, the Blue Nile samples (Figure 12) exhibited a denser correlation structure, characterized by stronger positive associations among Fe, Al, Ti, V, Cr, and Cu, as well as correlations with Ca, Mn, and Co. Although the overall organization of the correlation network was similar to that observed in Kordofan, several pairs of elements differed in both the magnitude and direction of their correlations. These correlative patterns are vital for understanding mineral co-occurrence in baobab bark.

### 3.5. Principal Component Analysis (Exploratory Analysis)

**Figure 14:**
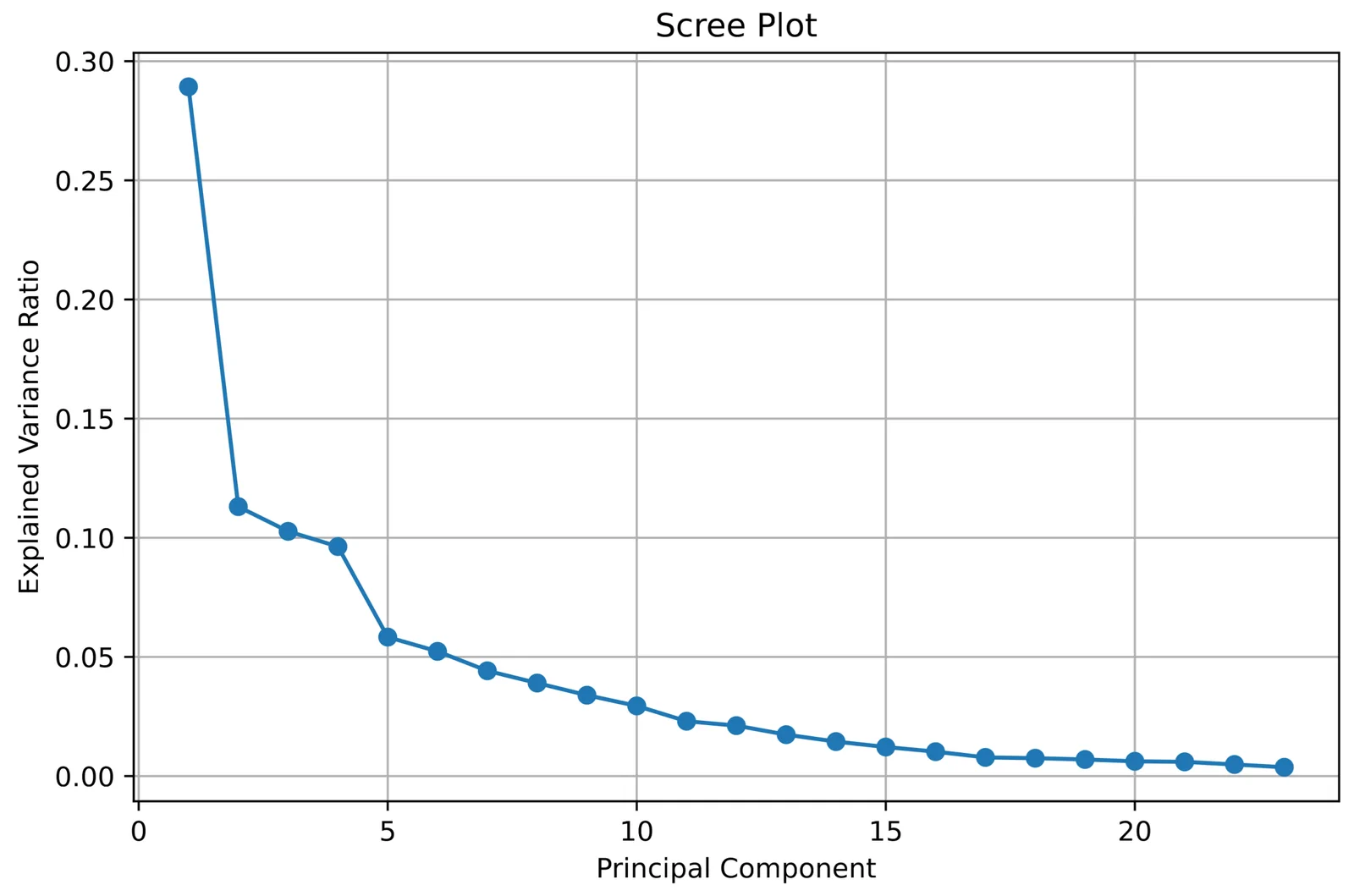
Scree plot for principal component analysis (PCA) of nutritional components in baobab bark.

Principal component analysis (PCA) was performed as an exploratory multivariate technique to visualize patterns in the elemental composition of baobab bark samples after variable standardization. The scree plot in Figure 14 suggests that several principal components contributed to the overall variability; however, only the first two principal components were retained for visualization because they explained the largest proportion of the variance (PC1 = 31.1%, PC2 = 15.0%), accounting for 46.1% of the total variability.

**Figure 15:**
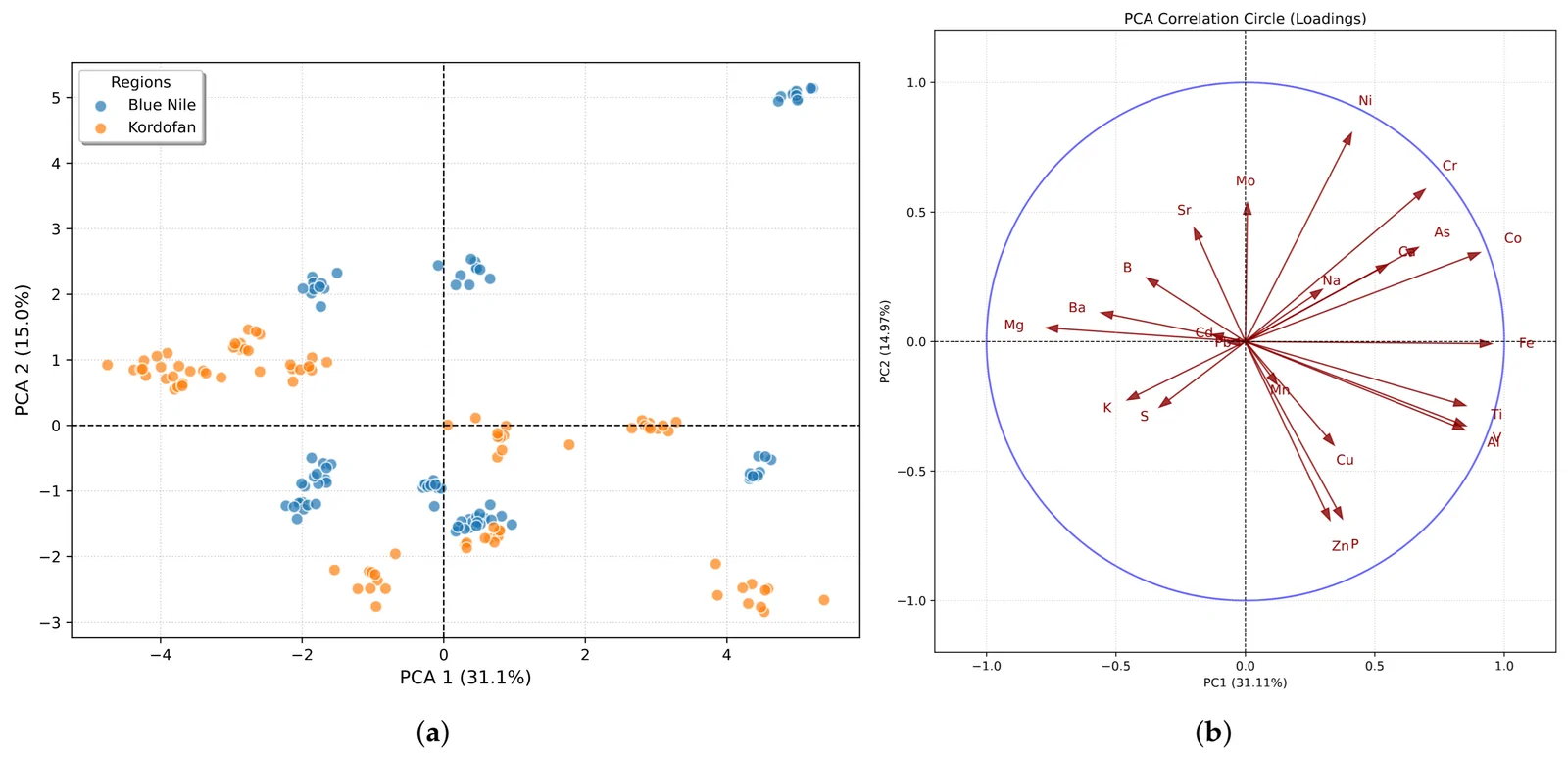
Principal component analysis (PCA) of the elemental composition of *Adansonia digitata* bark. (**a**) PCA score plot showing the distribution of samples from the Blue Nile (blue points) and North Kordofan (orange points) regions. (**b**) PCA loading plot illustrating the contribution of the analyzed elements to the first two principal components. The blue circle represents the correlation (unit) circle, indicating the maximum possible correlation with the principal components; variables located closer to the circle are better represented by PC1 and PC2, while the vectors’ length and direction indicate the strength and direction of their associations with the principal components.

**Figure 16:**
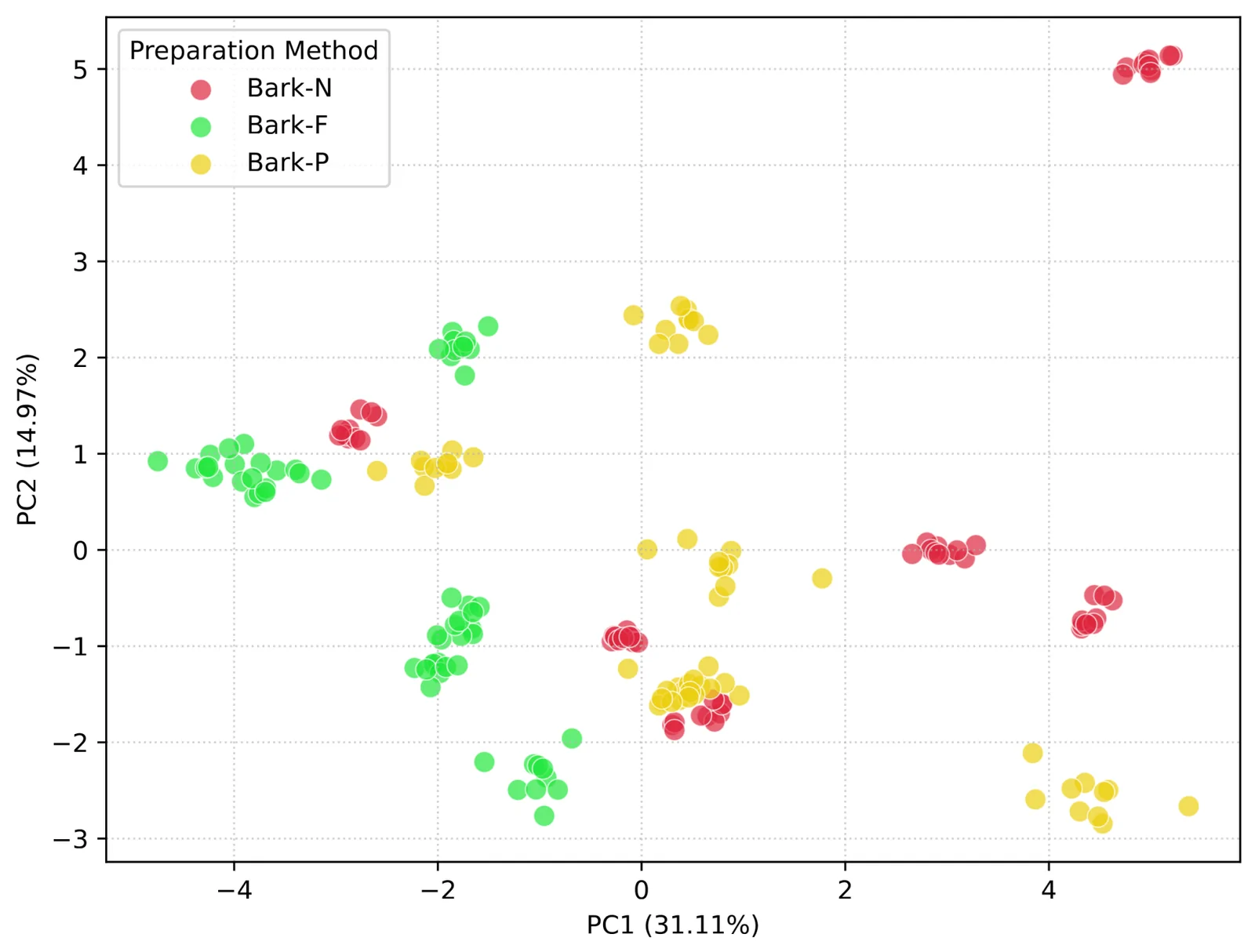
Score plot for sample preparation.

The PCA score plots in Figure 15a and Figure 16 reveal partial clustering according to both region and sample preparation, although substantial overlap among groups remained, indicating that these factors explain only part of the multivariate variability in elemental composition. Samples from the Blue Nile and Kordofan regions showed moderate separation primarily along PC1, whereas Bark-F samples tended to occupy the negative side of PC1, and Bark-N and Bark-P samples exhibited greater overlap around the centre and in the positive direction of the axis.

The loading plot in Figure 15b indicates that Fe, Al, Ti and V contributed strongly to the positive direction of PC1, whereas Mg, Ba and Sr were associated with the negative direction of the same component, reflecting their major contributions to the observed multivariate variation. Because PCA is an exploratory dimensionality-reduction technique rather than a statistical hypothesis test, the observed clustering should be interpreted descriptively. Statistical evidence for the effects of region and sample preparation is provided by the linear mixed-effects models, whereas pairwise relationships among elements are presented separately using Spearman’s rank correlation analysis.

## 4. Discussion

### 4.1. Regional Variability in Elemental Composition and Implications for Elemental Safety Screening

This study revealed clear regional differences in the elemental composition of *Adansonia digitata* bark collected from the Blue Nile and North Kordofan regions of Sudan. Similar regional variation in the mineral composition of plant materials has been reported previously and is commonly associated with differences in growing environments, although the present study did not investigate the environmental factors responsible for these differences [21,22,23]. The observed variation highlights the importance of accounting for geographical origin when characterizing the elemental composition of baobab bark and when comparing results across studies. The present study also demonstrated that elemental distribution differed among the three bark sample forms, indicating that the physical separation of bark tissues influences the distribution of several mineral elements. These findings provide baseline compositional data for baobab bark and contribute to a better understanding of its elemental profile [24]. The measured concentrations of selected potentially toxic elements were generally low and provide preliminary information for elemental safety screening.

### 4.2. Influence of Bark Sample Form on Elemental Distribution

Although all samples originated from the same plant organ, the elemental composition differed among the three bark sample forms. These differences most likely reflect the heterogeneous anatomical structure of baobab bark and the redistribution of tissues during mechanical fractionation rather than changes in elemental composition caused by the grinding process itself [25,26,27]. Whole bark (Bark-N), the powder fraction (Bark-P), and the fibre fraction (Bark-F) exhibited distinct elemental profiles, with several essential and other naturally occurring elements showing preferential distribution among the fractions. In contrast, the concentrations of selected potentially toxic elements were generally low across all bark sample forms, although significant differences were observed for specific elements such as Cr and As. These findings demonstrate that the bark sample form should be considered when characterizing the elemental composition of baobab bark. Standardized sample preparation procedures are therefore important for improving the comparability of analytical results across studies and supporting future nutritional, pharmaceutical, and industrial investigations [28].

## 5. Conclusions

This study provides one of the first comprehensive characterizations of the elemental composition of *Adansonia digitata* bark collected from two regions of Sudan. Significant differences were observed in the concentrations of several essential macroelements, essential trace elements, other naturally occurring elements, and selected potentially toxic elements among regions and bark sample forms highlighting that baobab bark from the Blue Nile region generally contained higher concentrations of K, P, Fe, Cu, Co, and Mo, whereas bark from North Kordofan was characterized by higher levels of Mg, Na, Mn, Zn, Ba, and Sr. The comparison of whole bark, powder, and fibre fractions further demonstrated that elemental distribution varied with bark sample form. The measured concentrations of selected potentially toxic elements provide preliminary baseline data for elemental safety screening but should not be interpreted as a comprehensive safety assessment. Overall, the findings contribute new information on the elemental composition of baobab bark and provide a scientific basis for future nutritional, pharmaceutical, and industrial research. Further studies should investigate elemental speciation, bioaccessibility, bioavailability, dietary exposure, and environmental factors to better understand the nutritional significance and safety of baobab bark.

### Strengths and Limitations

This study establishes baseline data for an underexplored plant material of ethnomedicinal importance. Using ICP-OES, the study quantified essential macroelements, essential trace elements, other naturally occurring elements, and selected potentially toxic elements, providing new insights into the distribution of mineral elements and supporting future research in nutrition, pharmaceuticals, and industry.

The study measured total elemental concentrations and therefore provides a preliminary safety screening rather than a comprehensive safety assessment. The exact amount of bark removed from each tree and the proportion (yield) of the powder and fibre fractions were not recorded. Elemental speciation, bioaccessibility, bioavailability, and dietary exposure were not evaluated. In addition, soil properties and other environmental factors were not investigated; therefore, the observed regional differences should be interpreted as location-specific observations rather than evidence of causal environmental effects. Future studies should integrate these approaches to provide a more comprehensive evaluation of the nutritional significance and safety of baobab bark.

## Figures and Tables

**Table 1:** Median (IQR) of elemental concentrations (ppm) of Baobab Bark by region (Blue Nile and Kordofan Regions in Sudan), all bark sample forms combined.

Category	Element	Blue Nile (Median ± IQR)	Kordofan (Median ± IQR)
Essential Macro Elements	Ca	40,900.0 ± 13,675.0	38,125.0 ± 11,207.5
	K	12,810.0 ± 2927.5	11,350.0 ± 825.0
	Na	51.07 ± 25.23	57.95 ± 19.7
	Mg	3849.5 ± 801.0	4596.5 ± 1610.75
	P	709.5 ± 289.75	339.0 ± 918.75
	S	545.0 ± 152.25	470.0 ± 129.25
Essential Trace Elements	Fe	228.0 ± 204.25	159.0 ± 216.56
	Mn	13.9 ± 8.75	27.24 ± 39.67
	Zn	9.05 ± 4.32	12.9 ± 5.62
	Cu	6.79 ± 4.71	3.18 ± 1.48
	Co	0.26 ± 0.13	0.14 ± 0.17
	Mo	0.23 ± 0.2	0.1 ± 0.02
Naturally occurring elements	B	15.95 ± 2.44	14.1 ± 2.18
	Sr	211.0 ± 45.75	236.5 ± 90.25
	Ba	67.0 ± 37.5	107.0 ± 141.5
	Ni	0.94 ± 2.95	1.31 ± 0.16
	Ti	1.88 ± 1.04	1.85 ± 3.12
	V	0.3 ± 0.41	0.2 ± 0.36
	Al	219.0 ± 220.5	152.5 ± 178.58
Potentially Toxic elements	Pb	0.29 ± 0.07	0.3 ± 0.12
	Cd	0.05 ± 0.04	0.06 ± 0.03
	Cr	0.69 ± 0.49	0.45 ± 0.25
	As	0.09 ± 0.02	0.09 ± 0.02

**Table 2:** Elemental composition (ppm, median ± IQR) of *Adansonia digitata* bark from the Blue Nile and North Kordofan regions of Sudan, prepared as three bark sample forms: Bark-N, Bark-F, and Bark-P.

Category	Element	Bark-N (Median ± IQR)	Bark-F (Median ± IQR)	Bark-P (Median ± IQR)
Essential Macro Elements	Ca	40,430.0 ± 16,420.0	47,805.0 ± 12,445.0	32,400.0 ± 6097.5
	K	10,635.0 ± 1550.0	11,690.0 ± 1675.0	12,540.0 ± 1602.5
	Na	67.28 ± 14.1	48.1 ± 20.02	47.95 ± 10.42
	Mg	3556.0 ± 320.0	3917.5 ± 689.75	4550.0 ± 1127.5
	P	586.5 ± 529.75	711.5 ± 490.75	578.5 ± 407.75
	S	434.0 ± 164.75	530.5 ± 189.5	586.5 ± 221.0
Essential Trace Elements	Fe	261.7 ± 336.1	292.5 ± 113.0	72.75 ± 66.35
	Mn	21.16 ± 11.31	24.95 ± 34.45	17.95 ± 8.42
	Zn	9.79 ± 6.29	12.6 ± 7.05	9.49 ± 4.77
	Cu	4.0 ± 3.56	4.18 ± 4.66	3.39 ± 2.07
	Co	0.29 ± 0.23	0.28 ± 0.09	0.11 ± 0.04
	Mo	0.14 ± 0.12	0.12 ± 0.13	0.12 ± 0.19
Naturally occurring elements	B	14.14 ± 1.34	15.75 ± 2.12	15.3 ± 5.67
	Sr	210.5 ± 50.25	236.0 ± 35.75	196.0 ± 101.75
	Ba	83.5 ± 46.0	80.0 ± 34.25	71.0 ± 153.25
	Ni	1.27 ± 0.12	1.33 ± 0.84	1.23 ± 1.62
	Ti	2.54 ± 3.32	2.23 ± 1.7	1.06 ± 0.87
	V	0.41 ± 0.32	0.48 ± 0.34	0.09 ± 0.05
	Al	223.5 ± 65.55	252.5 ± 159.0	77.85 ± 44.3
Potentially Toxic Elements	Pb	0.3 ± 0.18	0.3 ± 0.1	0.29 ± 0.08
	Cd	0.06 ± 0.03	0.06 ± 0.03	0.06 ± 0.03
	Cr	0.59 ± 0.56	0.83 ± 0.83	0.2 ± 0.17
	As	0.12 ± 0.06	0.08 ± 0.02	0.08 ± 0.02

**Table 3:** Likelihood ratio tests (*p*-values) for the main effects of region, sample form, and their interaction on elemental concentrations.

Group	Element	Region	Sample Form	Region × Sample Form
**Essential Macro Elements**	Ca	0.943	**<0.001**	**0.005**
	K	**<0.001**	**<0.001**	**<0.001**
	Na	**<0.001**	**<0.001**	**<0.001**
	Mg	**<0.001**	**<0.001**	**<0.001**
	P	**0.002**	0.126	0.893
	S	0.236	**<0.001**	0.349
**Essential Trace Elements**	Fe	**<0.001**	**<0.001**	**<0.001**
	Mn	**<0.001**	**<0.001**	**0.003**
	Zn	**<0.001**	**<0.001**	**<0.001**
	Cu	**<0.001**	**<0.001**	0.164
	Co	**<0.001**	**<0.001**	**<0.001**
	Mo	**<0.001**	0.168	**0.002**
**Naturally occurring elements**	B	**<0.001**	**<0.001**	**<0.001**
	Ba	**<0.001**	0.197	0.109
	Sr	**0.005**	**<0.001**	**<0.001**
	Ni	**0.004**	**0.011**	**0.004**
	Ti	0.054	**<0.001**	**<0.001**
	V	**0.017**	**<0.001**	**0.004**
	Al	**<0.001**	**<0.001**	**0.002**
**Potentially Toxic elements**	Pb	0.061	0.089	**<0.001**
	Cd	0.804	0.999	0.970
	Cr	**<0.001**	**<0.001**	**0.047**
	As	0.053	**<0.001**	**0.017**

Note: Bold values indicate statistical significance (p≤0.05). Values reported as 0.000 by the statistical software are represented here as <0.001.

**Table 4:** Pairwise comparisons (*p*-values) among bark sample preparation forms (Bark-N, Bark-P, Bark-F) nested within the Blue Nile and North Kordofan regions.

		Blue-Nile			Kordofan		
**Group**	**Element**	**Bark-N vs. Bark-P**	**Bark-N vs. Bark-F**	**Bark-P vs. Bark-F**	**Bark-N vs. Bark-P**	**Bark-N vs. Bark-F**	**Bark-P vs. Bark-F**
**Essential Macro Elements**	Ca	**0.018**	**<0.001**	**<0.001**	**<0.001**	**<0.001**	**<0.001**
	K	**<0.001**	**<0.001**	**0.014**	**<0.001**	**<0.001**	**0.002**
	Na	**<0.001**	**<0.001**	0.240	**<0.001**	**<0.001**	**<0.001**
	Mg	**<0.001**	**<0.001**	0.096	**0.029**	**<0.001**	**<0.001**
	P	**0.019**	0.067	**<0.001**	0.408	0.846	0.542
	S	**<0.001**	**<0.001**	**0.043**	**<0.001**	**<0.001**	0.805
**Essential Trace Elements**	Fe	**0.012**	**<0.001**	**<0.001**	**<0.001**	**<0.001**	**<0.001**
	Mn	0.348	**<0.001**	**<0.001**	0.309	0.898	0.272
	Zn	**<0.001**	**<0.001**	**<0.001**	**0.016**	**0.026**	0.874
	Cu	0.896	0.074	0.099	0.209	**<0.001**	**<0.001**
	Co	**<0.001**	**<0.001**	**<0.001**	**0.004**	**<0.001**	**<0.001**
	Mo	0.678	**0.001**	**0.007**	0.773	0.293	0.182
**Naturally occurring elements**	B	**0.006**	0.573	**0.001**	**<0.001**	**<0.001**	**<0.001**
	Ba	0.335	0.963	0.308	0.318	**0.020**	0.204
	Sr	**<0.001**	**<0.001**	**<0.001**	**0.001**	**0.004**	0.739
	Ni	0.344	**0.002**	**0.035**	**<0.001**	0.223	**0.004**
	Ti	**<0.001**	**<0.001**	**<0.001**	**<0.001**	**<0.001**	**<0.001**
	V	**0.047**	**<0.001**	**<0.001**	**0.019**	**<0.001**	**<0.001**
	Al	0.600	**<0.001**	**<0.001**	**<0.001**	**<0.001**	**<0.001**
**Potentially Toxic elements**	Pb	**0.006**	**0.003**	0.932	**<0.001**	0.329	**0.004**
	Cd	0.976	0.932	0.910	0.973	0.834	0.852
	Cr	0.193	**<0.001**	**<0.001**	**0.033**	**<0.001**	**<0.001**
	As	**<0.001**	**<0.001**	0.592	0.184	0.205	0.936

Note: Bold values indicate statistical significance (p≤0.05). Values reported as 0.000 by the statistical software are represented here as <0.001.

**Table 5:** Simple main effects (*p*-values) isolating the significance of regional differences (Blue Nile vs. Kordofan) strictly within each specific bark sample form.

Group	Element	Bark-N	Bark-P	Bark-F
		Blue Nile vs. Kordofan	Blue Nile vs. Kordofan	Blue Nile vs. Kordofan
**Essential Macro Elements**	Ca	**0.016**	0.953	**0.026**
	K	0.092	**<0.001**	0.837
	Na	0.323	**<0.001**	0.098
	Mg	**<0.001**	0.993	**<0.001**
	P	**0.045**	**0.045**	0.152
	S	0.808	0.928	0.067
**Essential Trace Elements**	Fe	**<0.001**	0.326	**<0.001**
	Mn	**0.001**	**<0.001**	**<0.001**
	Zn	0.162	0.191	**<0.001**
	Cu	**<0.001**	**<0.001**	**<0.001**
	Co	**<0.001**	0.460	**0.017**
	Mo	**<0.001**	**<0.001**	**<0.001**
**Naturally occurring elements**	B	**<0.001**	**<0.001**	**0.007**
	Ba	**<0.001**	**<0.001**	**<0.001**
	Sr	0.134	0.297	**<0.001**
	Ni	0.415	**0.043**	**<0.001**
	Ti	**<0.001**	**<0.001**	**<0.001**
	V	**0.003**	0.195	**0.010**
	Al	**<0.001**	0.632	**0.001**
**Potentially toxic elements**	Pb	**0.025**	**<0.001**	0.164
	Cd	0.956	0.975	0.738
	Cr	**<0.001**	**<0.001**	**<0.001**
	As	**0.001**	0.979	0.952

Note: Bold values indicate statistical significance (p≤0.05). Values reported as 0.000 by the statistical software are represented here as <0.001.

## Data Availability

The original contributions presented in this study are included in the article. Further inquiries can be directed to the corresponding authors.

## Appendix A

**Table A1:** Analytical Quality Assurance and Quality Control (QA/QC) Parameters for ICP-OES Determination of Elements in Baobab Bark.

Element	Wavelength (nm)	Cal. Range (mg L_−1_)	Calibration (*R*^2^)	LOD (mg L_−1_)	LOQ (mg L_−1_)	Procedural Blank (mg L_−1_)
Ca	422.673	2–1000	0.9985	1.1125	3.5	<LOD
K	766.490	2–1000	1.0000	0.1370	0.50	<LOD
Na	589.592	0.4–200	1.0000	0.6100	2.0	<LOD
Mg	279.553	1–500	0.9995	0.2550	0.50	<LOD
P	178.287	0.4–200	0.9976	0.0130	0.080	<LOD
S	180.734	0.4–200	0.9974	1.2500	3.5	<LOD
Fe	259.940	0.1–50	1.0000	1.0100	3.0	<LOD
Mn	257.610	0.04–20	1.0000	0.0020	0.050	<LOD
Zn	213.856	0.012–6	1.0000	0.1140	0.400	<LOD
Cu	324.754	0.004–2	1.0000	0.0340	0.100	<LOD
Co	228.616	0.001–0.5	0.9998	0.0300	0.050	<LOD
Mo	202.032	0.004–2	1.0000	0.0240	0.080	<LOD
B	249.773	0.005–2.5	1.0000	0.0680	0.200	<LOD
Ba	455.404	0.01–5	0.9999	0.0040	0.050	<LOD
Sr	407.771	0.012–6	1.0000	0.0040	0.100	<LOD
Ni	231.603	0.002–1	0.9999	0.0540	0.200	<LOD
Ti	337.279	0.004–2	1.0000	0.0050	0.100	<LOD
V	292.400	0.001–0.5	1.0000	0.0020	0.090	<LOD
Al	237.312	0.1–50	0.9999	0.7670	1.0	<LOD
Pb	220.351	0.004–2	0.9999	0.0530	0.080	<LOD
Cd	226.502	0.002–1	0.9999	0.0120	0.050	<LOD
Cr	267.716	0.001–0.5	0.9999	0.0050	0.050	<LOD
As	189.042	0.01–5	0.9998	0.1140	0.250	<LOD

**Table A2:** Validation of ICP-OES accuracy using certified and assigned reference values.

Element	Reference Value (mg kg^−1^)	Source	Measured Value (mg kg^−1^)	Recovery (%)	Assessment
Ca	18,500	IPE/WEPAL assigned value	18,770	101.46	Acceptable
K	21,900	IPE/WEPAL assigned value	20,240	92.42	Acceptable
Na	65.5	IPE/WEPAL assigned value	64.01	97.73	Acceptable
Mg	2610	IPE/WEPAL assigned value	2577	98.74	Acceptable
P	2600	IPE/WEPAL assigned value	2520	96.92	Acceptable
S	2100	IPE/WEPAL assigned value	2264	107.81	Acceptable
Fe	132	IPE/WEPAL assigned value	126.4	95.76	Acceptable
Mn	16.8	ERM^®^-CD281 certified value	15.78	93.93	Acceptable
Zn	45.0	ERM^®^-CD281 certified value	45.6	101.33	Acceptable
Cu	11.2	ERM^®^-CD281 certified value	10.897	97.29	Acceptable
Co	0.075	IPE/WEPAL assigned value	0.081	108.00	Acceptable
Mo	0.135	ERM^®^-CD281 certified value	0.122	90.37	Acceptable
B	34.3	ERM^®^-CD281 certified value	32.93	96.01	Acceptable
Ba	54.7	IPE/WEPAL assigned value	55.39	101.26	Acceptable
Sr	60.2	IPE/WEPAL assigned value	58.22	96.71	Acceptable
Ni	1.00	ERM^®^-CD281 certified value	0.974	97.40	Acceptable
Ti	Not available	No reference value	0.333	N/A	Reference value unavailable
V	0.210	IPE/WEPAL assigned value	0.219	104.29	Acceptable
Al	107	IPE/WEPAL assigned value	97.43	91.06	Acceptable
Pb	2.08	ERM^®^-CD281 certified value	2.18	104.81	Acceptable
Cd	0.0215 *	ERM^®^-CD281 certified value	<0.050	N/A	Below reporting limit
Cr	1.38	ERM^®^-CD281 certified value	1.27	92.03	Acceptable
As	0.0707 *	ERM^®^-CD281 certified value	<0.250	N/A	Below reporting limit

Abbreviations: IPE, International Plant-Analytical Exchange; WEPAL, Wageningen Evaluating Programs for Analytical Laboratories; CRM, Certified Reference Material. 1. Certified reference values were obtained from the ERM®-CD281 (Rye Grass) Certified Reference Material for elements with certified concentrations. 2. Assigned reference values for elements not certified in ERM®-CD281 (Ca, K, Na, Mg, P, S, Fe, Co, Ba, Sr, V and Al) were obtained from the International Plant-Analytical Exchange (IPE/WEPAL) proficiency testing scheme, which provides robust consensus (assigned) values derived from inter-laboratory measurements. 3. Recovery (%) was calculated as: Recovery (%) = $\frac{\mathrm{Measured}\;\mathrm{value}}{\mathrm{Reference}\;\mathrm{value}}\times 100$. 4. For Cd and As, the laboratory reporting limits exceeded the reference concentrations; therefore, recovery could not be reliably calculated, and these elements are reported as N/A (Below reporting limit). 5. Ti is not included in either the ERM®-CD281 certification or the IPE assigned values; therefore, no recovery was calculated. 6. * Reference value available; however, the measured concentration was below the laboratory reporting limit, and therefore recovery was not calculated.
